# Small supernumerary marker chromosomes derived from human chromosome 11

**DOI:** 10.3389/fgene.2023.1293652

**Published:** 2023-12-15

**Authors:** Thomas Liehr, Monika Ziegler, Luisa Person, Stefanie Kankel, Niklas Padutsch, Anja Weise, Jörg Paul Weimer, Heather Williams, Susana Ferreira, Joana B. Melo, Isabel M. Carreira

**Affiliations:** ^1^ Institute of Human Genetics, Jena University Hospital, Friedrich Schiller University, Jena, Germany; ^2^ Department of Gynecology and Obstetrics, University Hospital of Schleswig-Holstein, University Kiel, Kiel, Germany; ^3^ Cache DNA, Inc., San Carlos, CA, United States; ^4^ Cytogenetics and Genomics Laboratory, CACC, iCBR/CIMAGO, CIBB, Faculty of Medicine, University of Coimbra, Coimbra, Portugal

**Keywords:** small supernumerary marker chromosomes (sSMCs), chromosome 11, triplosensitive genes, pericentric region, copy number variation (CNV), uniparental disomy (UPD), imprinting

## Abstract

**Introduction:** With only 39 reported cases in the literature, carriers of a small supernumerary marker chromosome (sSMC) derived from chromosome 11 represent an extremely rare cytogenomic condition.

**Methods:** Herein, we present a review of reported sSMC(11), add 18 previously unpublished cases, and closely review eight cases classified as ‘centromere-near partial trisomy 11’ and a further four suited cases from DECIPHER.

**Results and discussion:** Based on these data, we deduced the borders of the pericentric regions associated with clinical symptoms into a range of 2.63 and 0.96 Mb for chromosome 11 short (p) and long (q) arms, respectively. In addition, the minimal pericentric region of chromosome 11 without triplo-sensitive genes was narrowed to positions 47.68 and 60.52 Mb (GRCh37). Furthermore, there are apparent differences in the presentation of signs and symptoms in carriers of larger sSMCs derived from chromosome 11 when the partial trisomy is derived from different chromosome arms. However, the number of informative sSMC(11) cases remains low, with overlapping presentation between p- and q-arm-imbalances. In addition, uniparental disomy (UPD) of ‘normal’ chromosome 11 needs to be considered in the evaluation of sSMC(11) carriers, as imprinting may be an influencing factor, although no such cases have been reported. Comprehensively, prenatal sSMC(11) cases remain a diagnostic and prognostic challenge.

## 1 Introduction

A small supernumerary marker chromosome (sSMC) is a cytogenomic condition based on the presence of a numerical and structural aberration. In most cases, an sSMC, which can derive from any human chromosome, is present in addition to a normal karyotype of 46, XX or 46,XY. sSMCs are found in 0.044% of the human population, with ∼3.5 million sSMC carriers among the present population of ∼8 billion. Approximately 2.5 million (70%) sSMC carriers are clinically normal and located through chromosomal analyses for a different reason for referral. The remaining 30% have mild to severe clinical signs and symptoms due to the sSMC. Parental inheritance accounts for 30% and ∼50% of sSMC carriers are mosaic; mosaicism may also be ‘cryptic’, which means that molecular cytogenetic studies reveal submicroscopic differences in sSMC shape and content (Liehr, 2012; Liehr, 2023).

Using size, sSMCs are defined as generally smaller than a chromosome 20 within the same metaphase spread and can take different shapes, i.e., ring- (r), inverted duplication- (inv dup), or ‘centric-minute’- (min) shaped. Mitotic instability is elevated in min- and r-shaped sSMCs compared to inv-dup-shaped; thus, the former are more likely to lead to mosaicism (Hussein et al., 2014). In addition, sSMCs may be composed of material from a single chromosome or two or more chromosomes–the latter creates ‘complex sSMCs’ (Liehr et al., 2013). Suppose the sSMC material is derived from a single chromosome, but the order of the DNA stretches is disrupted or rearranged compared to a normal sister chromosome; in this case, it is termed ‘discontinuous sSMC’. These discontinuous sSMCs most likely form from a chromothripsis-related trisomic rescue event (Kurtas et al., 2019). In addition, an sSMC can carry no normal centromeric region but instead carry a neocentromere (Liehr et al., 2007). Finally, an sSMC can co-occur with uniparental disomy (UPD) of the normal sister chromosomes; co-occurrence accounts for ∼3% of *de novo* cases and ∼1.3% of all reported cases (Liehr et al., 2011). Patients may carry 2 to 7 sSMCs derived from different chromosomes or multiple sSMCs, which accounts for ∼1.2% of all cases (Robberecht et al., 2012). In sSMC cases with UPD, heterochromatic sSMCs not resulting in relevant copy number variations (CNVs) may still be causative for clinical presentation, either because (i) they carry genes involved with imprinting, and/or (ii) there is a (partial) isodisomy that leads to the activation of a parental recessive gene variant (Benn, 2021). Interestingly, in rare cases, mosaicism may also influence the phenotype (Liehr and Al-Rikabi, 2019).

Of the estimated 3.5 million sSMC carriers currently in the population, only ∼7,169 are documented in the literature (Liehr, 2023). The shape is not reported for 852 cases (∼12%), and 138 cases (∼2%) are neocentric. Of the remaining 6,179 reported cases, 3,795 (∼62%) have inv dup-, 1,563 (∼25%) min-, and 821 (∼13%) r-shaped (Liehr, 2023).

Few sSMCs are specifically attributed to a clinical syndrome, as outlined below (Liehr, 2023):

• +inv dup(5)(pter→q10::q10→pter) / tetrasomy 5p-syndrome (Blakey-Cheung et al., 2020, ORPHA:3309);
• +inv dup(8)(pter→q10::q10→pter) / tetrasomy 8p-syndrome (Napoleone et al., 1997, ORPHA:3307, OMIM #614290);
• +inv dup(9)(pter→q10∼12::q10∼12→pter) / tetrasomy 9p-syndrome (Süleyman et al., 2022, ORPHA:3310);
• +inv dup(12)(pter→q10∼12::q10∼12→pter) / tetrasomy 12p- or Pallister-Kilian-syndrome (ORPHA:884, OMIM #601803);
• +der(13 or 21)t(13 or 21;18)(q11.1;p11.1) / special form of trisomy 18p-syndrome (Liehr et al., 2013);
• +inv dup(15)(pter→q12∼13::q12∼13→pter) / proximal tetrasomy 15q-syndrome (Battaglia, 2008, ORPHA:3306);
• +inv dup(18)(pter→q10::q10→pter) / tetrasomy 18p-syndrome (ORPHA:3307, OMIM #614290);
• +inv dup(20)(pter→q10::q10→pter) / tetrasomy 20p-syndrome (Maziad and Seaver, 2015);
• +inv dup(22)(pter→q11.2::q11.2→pter) / proximal tetrasomy 22q- or cat eye-syndrome (ORPHA:195, OMIM #115470);
• +der(22)t(8;22)(q24;q11.1∼11.2) / supernumerary der(22)t(8;22)-syndrome (OMIM #613700);
• +der(22)t(11;22)(q23;q11.2) / derivative chromosome 22- or Emanuel-syndrome (ORPHA:96170, OMIM #609029).

These 11 syndromes constitute ∼34% of the reported cases. In addition, there are 786 sSMC cases reported in Turner syndrome mosaics (∼11%) (Li et al., 2021; Liehr, 2023).

For all other sSMCs, chromosome-specific genotype–phenotype correlations are based only on a paucity of cases. However, more and more evidence has emerged that proximal centromere-near regions of each human chromosome mainly carry triplo-insensitive genes (Liehr, 2023). Thus, in many cases, a proximal partial trisomy leads to no clinical symptoms; these sSMCs may be transmitted through generations but may adversely affect fertility, mainly in male carriers (Dalprà et al., 2005). Still, in sSMC cases comprised of centromere-near material distal to a specific region, the presence of triplo-sensitive genes leads to clinical signs in the carrier. Phenotypic effects can be chromosome-arm-specific differently, e.g., for cases with sSMC-induced proximal partial trisomy 11p or 11q.

The available data for triplo-sensitive regions is summarized herein, and a possible first genotype-phenotype correlation for sSMCs derived from proximal 11p and/or 11q is presented. Please note that sSMCs are grouped according to the origin of their centromere; thus, Emanuel syndrome cases with +der(22)t(11; 22) are not included (OMIM #609029).

## 2 Material and methods

### 2.1 Literature search and patients studied

All 57 sSMC cases were either studied in the laboratory of TL (Jena, Germany–for contributors see also in Acknowledgments) and/or previously published; they are also recorded in the sSMC database (Liehr, 2023). Herein, all 39 previously (by several authors) published sSMC(11) cases and 18 new cases studied in Jena are included (see Supplementary Table S1). The study’s own cases were acquired through routine diagnostic testing and patients were anonymized. Written informed consent was obtained from the individual(s) or minors’ legal guardian/next of kin for the publication of any potentially identifiable data included in this article. In addition, ethical approval was provided by the Ethical Commission of Jena University Hospital (code 4738-03/16) for this sSMC research. Lastly, eight cases from the literature with copy number gains near centromere 11 were also collected and summarized (see Supplementary Table S2). In total, 65 cases are listed in Tables 1, 2, with minimal clinical information and molecular(cyto)genetic study results–more data is provided in the corresponding Supplementary Tables S1, S2.

**TABLE 1:** Overview of the 57 sSMC(11) cases as characterized at present—for more details, see Supplementary Table S1; all molecular karyotyping data is given here in build GRCh37/hg19.

#	Karyotype	Ref
sSMC(11) cases: no clinical findings		
1	47,XY,+r(11)(::p11.12→q12.2::)	Liehr, 2014 case 11-1
	.arr[hg19] 11q12.1-12.2(51,095,992_60,473,821)x3	
2	47,XY,+mar mat.ish min(11)(:p11.12→q11:)	this study
	(RP11-397M16+,D11Z1+,RP11-77M17-)	
3	mos 47,XY,+mar[50%]/46,XY[50%].ish min(11)(:p11.1→q11:)	this study
	(RP11-397M16-,D11Z1+,RP11-77M17-)	
4	mos 47,XY,+mar[21]/46,XY[32].ish min(11)(:p11.1→q11:)	this study
	(RP11-397M16-,D11Z1+,RP11-77M17-)	
5	mos 47,XX,+mar[18]/46,XX[4].ish min(11)(:p11.1→q11:)	Chen et al. (2017)
	(D11Z1+).arr[hg18] (X,1-22)x2	
6	47,XX,+mar.ish r(11)(::p11.1→q12.2::)[10]/r(11)(::p11.1→q12.2:	this study
	:q12.2→p11.1::)[7]/min(:q12.2→p11.1::p11.1→q12.2:)[3] (RP11-397M16-,D11Z1+,RP11-77M17+)	
7	47,XY,+mar.ish min(11)(:p11.1→q12.1:)(RP11-397M16-,D11Z1+,RP11-77M17+)	this study
	.arr[hg19] 11q12.1(55,896,790_59,319,390)x3	
8	mos 47,XX,+mar pat[60%]/46,XX[40%].ish r(11)(D11Z1+)	Haaf et al. (1992)
9	mos 47,XY,+mar[32]/46,XY[12].ish r(11)(wcp11+)	Kozlowski et al., 2006 case 26
**sSMC(11) cases: with clinical findings**		
10	mos 47,XN,+mar[74%]/46,XN[26%].arr[hg19] 11p12∼11.2(42,922,228_50,768,675)x3	Joshi et al., 2019 case P10
11	mos 47,XX,+mar[53%]/46,XX[47%]	Neill et al., 2010 case 29361
	aCGH data details not provided	
12	mos 47,XY,+mar[77%]/46,XY[23%].ish min or r(11)(:p11.2→q11.1:)(RP11-397M16+,D11Z1+,RP11-77M17-)	this study
13	mos 47,XY,+mar[33%]/46,XY[67%].ish min(11)(:p12→q11:)(RP11-397M16+,D11Z1+,RP11-77M17-)	Guilherme et al., 2012case Sm-5
	.arr[hg18] 11p12(40,190,000_54,700,000)x3	
14	mos 47,XN,+mar[14%]/46,XN[86%]	Baldwin et al., 2008 case 13
	most likely a r(11)(::p11.12→q12.1::); no clear data for aCGH - only given: size on sSMC p-arm 0.2 MB and q-arm 2.3 MB	
15	mos 47,XX,+mar mat[70%]/46,XX[30%].ish r(11)(::p11.12→q13.1::)[6]/r(11; 11)(::p11.12→q13.1:	this study
	:p11.12→q13.1::)[3]/min(11)(:p11.12→q13.1:)[4] (RP11-397M16+,D11Z1+,RP11-77M17+)	
	.arr[hg18] 11p11.12q13.1(50,470,000_65,020,000)x3	
16	mos 47,XY,+mar[86]/46,XY[14].ish r(11)(::p11.12→q13.1::)(RP11O-318O24+,RP11-100E23+,CTD-3202L3+,RP11-720L5+)	Castronovo et al., 2013 case 5
	no clear data for aCGH - only given: size on sSMC p-arm ∼1.5 Mb and q-arm 10.04 Mb	
17	47,XY,+mar[100%]	this study
18	mos 47,XX,+mar[?]/46,XX[?].ish min(11)(D11Z1+)	Rauch et al., 1992 case 7
19	AF: mos 47,XY,+mar[52%]/46,XY[48%]	Daniel and Malafiej 2003 case 7
	PBL at birth: 46, XY [200]	
	PBL at 4 m: mos 47,XY,+mar[36%]/46,XY[64%]	
	.ish r(11)(D11Z1+) no data for aCGH provided	
20	mos 48,XY,+mar1,+mar2[?]/47,XY,+mar1[?]/47,XY,+mar2[?]/46,XY[?]	Sanz et al., 2005 case 4
	.ish mar(11)(D11Z1+)	
21	mos 47,XY,+mar[70%]/46,XY[30%]	Silveira-Santos et al. (2012)
	no clear data for aCGH - only given: size 5.9 Mb	
**sSMC(11) cases without clinical details/clear clinical correlation**		
22	mos 47,XY,+mar[31]/46,XY[5].ish r(11)(::p11→q12::)(D11Z1+)	Leung et al., 2004 case 4
23	47,XN + mar[?%].ish mar(11)(D11Z1+)	Sanz et al., 2005 1 case
24	mos 47,XN + mar[50%]/46,XN[50%].ish r(11)(::p11→q12::)[3]/r(11; 11)(::p11→q12::p11→q12::)[2](RP11-397M16-,D11Z1+,RP11-77M17+)	this study
25	mos 47,XN,+mar(11)[70%]/46,XN[30%].ish min(11)(:p11.1→q11:)(RP11-397M16-,D11Z1+,RP11-77M17-)	this study
26	47,XX,+mar[100%].ish min(11)(:p11.1→q11:)(RP11-397M16-,D11Z1+,RP11-77M17-)	this study
27	mos 47,XX,+mar[21]/46,XX[30].arr[hg18] 11p12(43,085,000_51,400,000)x3	data provided by Dr. Joleen Viront, Akron, OH, United States
28	mos 47,XY,+mar(11)[10]/46,XY[5]	Thangavelu et al. (2011)
29	mos 47,XX,+mar[60%-90%]/46,XX[10%-40%].ish	Hamid et al., 2012 case 1
	min(11)(:p11.21→q13.1:)(RP11-397M16+,D11Z1+,RP11-77M17+)	
	.arr[hg19] 11p11.21q13.1(49,850,000_64,600,000)x3	
30	mos 47,XY,+mar[15]/46,XY[17].ish min(11)(:p11.?1→q1?1:)(RP11-397M16-,D11Z1+,RP11-77M17-)	this study
31	mos 47,XX,+mar[94]/46,XX[53].arr[hg18] 11q12.1q12.3(55,509,438_62,106,928)x3	Marle et al., 2014 case 13
32	mos 47,XX,+mar[33]/46,XX[25].arr[hg18] 11p13q12.1(34,890,001_56,410,001)x3	Malvestiti et al., 2014 case AF-11
33	mos 47,XX,+mar[71]/46,XX[22].ish min(11)(:p11.1→q12.1:)[3]/r(11)(::p11.2→q12.1::)[2]/r(11; 11)(::p11.2→q12.1::p11.2→q12.1::)[3](RP11-397M16+,D11Z1+,RP11-77M17+)	this study
34	mos 47,XY,+mar[2]/46,XY[48].ish min(11)(:p11.11→q11:)(RP11-397M16-,D11Z1+,RP11-77M17-)	this study
35	mos 47,XY,+mar[20%]/46,XY[80%].arr[hg19] 11p14.q12.1(30,800,000_56,650,000)x3	this study
36	mos 47,XX,+mar[38]/46,XX[12].ish min(11)(:p11.11→q11:)(RP11-397M16-,D11Z1+,RP11-77M17-)	this study
37	mos 47,XX,+mar[73]/46,XX[12].arr[hg19] 11p12.1q13.2(55,084,040_66,490,712)x3	Huang et al., 2019 case 16/16
38	mos 47,XY,+min[15%]/46,XY[85%]	Kontodiou et al. (2018)
	arr[hg19] 11p14q12.1(30,796,545_56,649,983)x3	
**Complex sSMC(11) cases without clinical details/clear clinical correlation**		
39	mos 47,XX,+mar dn[13]/46,XX[10].rev ish r(11)t(11; 20)(::11p11.1→11q12.1::20q13.1?2→q13.32::)	this study
40	47,XY,t(11; 13)(q25; q14),+der(11)t(11; 13)(q25; q14)	Metay et al. (2011)
**Discontinuous sSMC(11) cases without clinical details/clear clinical correlation**		
41	mos 47,XY,+mar[18]/46,XY[2] r(11)(::p11.2→q13.1:	Starke et al. (2001)
	:q14::).rev ish r(11)(::p11.2→q12.3::q14::)	
	.arr(hg18) 11p11.2q12.3(42.070,000_60,600,000)x3	
42	AF: mos 47,XY,+mar[13]/46,XY[1]	Kurtas et al., 2019 case sSMC11
	CVS: mos 47,XY,+mar[31]/46,XY[3] seq[GRCh37] r(11)(::p11.2→q12.1::q12.1→q12.1::p15.5→p15.5::p15.4→p15.4::p11.2→p11.2::q12.1→q12.1::)	
	chr11:g[47963807_cen_57123447inv::34232223_34232229::34232469_34232519::CACAGCTATGAGA::57123447_chr11:57150478:	
	:TTTCCATTCCA::chr11:1791532_chr11:1831828::chr11:3681909_chr11:3826675::AGAGATGGAGCAAGCAATAGCAACTGCATA: :chr11:45940475_45998725::CACTGTAAATTGGG::47277430_47429775inv::chr11:57151476_57152981inv] chr11:g[57452438_57453327::57150508_57151481inv::57451445_57452437::57276408_57278946::18428101_18558839::57278947_57297284inv]	
**sSMC(11) formed by McClintock mechanism**		
43	mos 47,XX,del(11)(p15.1p11.1),+r(11)(p15.1p11.1)mat[70%]/46,XX,del(11)(p51.1p11.1)[30%]	Maggert and Karpen (2000)
44	mos 47,XX,del(11)(p14.3p11.2),+r(11)(::p14.3→ neo→ 11.12::)[16]/46,XX,del(11)(p.14.3p11.2)[4]	Wang et al. (2023)
45	47,XX,del(11)(p11.12p11.2),+r(11)(::p11.12→p11.2::)[100%]	Chuang et al. (2005)
**sSMC(11) formed by pseudo-McClintock mechanism**		
46	47,XY,del(11)(q22),+inv dup(11)(qter→q22::q22→qter)[100%]	Amor and Choo (2002)
47	47,XX,del(11)(q21),+inv dup(11)(q21)[100%]	Pai et al. (1981)
**sSMC(11) in multiple sSMC carriers**		
48	mos 48,XX,+mar1,+mar2[16%]/47,XX,+mar1[26%]/47,XX,+mar2[22%%]	Haddad et al. (1998)
	46,XX[36%]	
	mar1 = ish min(6)(:p11.2→q12:)(wcp6+,D6Z1+);	
	mar2 = min(11)(:p11.11→q11:)(D11Z1+,wcp11-)	
49	mos 48,XY,+mar1,+mar2[?]/47,XY,+mar1[?]/47,XY,+mar2[?]/46,XY[?]	Plattner et al. (1993)
	mar 1: ish mar(11)(D11Z1+), mar2: ?	
50	mos 48,+mar1,+mar2[36%]/47,+mar1[36%]/47,+mar2[28%]	Ballif et al., 2007 case 6
	mar1: arr[hg18] min(11)(:p10→q12.1:)(RP11-736I10+)	
	mar2: arr[hg18] min(17)(:p11.2→q10:)(RP11-64J19+)	
51	mos 47,XX,+mar1[60%]/46,XX[40%]	this study
	second sSMC not detected in cytogenetics but in FISH	
	final karyotype	
	48,XY,+r(4)(::p14→q12::),+min(11)(:p11.11→q11:)[5]/48,XY,+r(4; 4)(::p14→q12::p14→q12::),	
	+min(11)(:p11.11→q11:)[5]/47,XY,+min(11)(:p11.11→q11:)[10]	
52	mos 49,XY,+3mar[13]/48,XY,+2mar[22]/47,XY,+mar[23]/46,XY[2]	Kurtas et al., 2019 case sSMC8a
	r(4)(::p12→q12::)[?]	
	min(8)(:p11.21→q11.21::)[6]	
	min(8)(:p21.1→p12: :p11.21→q11.21:)[14]	
	40.08-53.56 MB (hg19) r(11)(::p11.12→q11.1::)[?]	
	acc. to NGS sSMC(8) is a	
	mar(8)(:p11.21→q11.23:	
	:q12.1q12::q12q12:) spanning chr8:g[:53561524::GCCCTAAGGAATCTCC:	
	:60002688_60002774::TGG:	
	:55759348_55759565:	
	:TGA​TGT​GTC​ACC​TTG​CTT​TTA​GAT​CTG​AAG​GTG​A:	
	:40082798:]	
53	mos 50,XY,+mar1, +mar2, +mar3, +mar4[16]/49,XY,+mar1, +mar2, +mar3[28]/48,XY,+mar1,+mar2[48]/47,XY,+mar1[8]	Fei et al., 2011 1 case
	mar 1 = min(19)	
	mar 2 = min(11)	
	mar 3 = mar1	
	mar 4 = der(11; 19)	
	#11: arr[hg18] 11p11.12q12.1(48845776-58751035)x3	
	#19: arr[hg18] 19p12q12(23364013-34857827	
	possibly r(11; 19): arr[hg18] 11q11q12.1(55,140,785-58,610,968)x3,19p12q12(23,967,466-24,006,230; 34,272,160-34,429,875)x3	
54	mos 51,XX,+5mar[?%]/50,XX,+4mar[majority]/49,XX,+3mar[?%]/48,XX,+2mar[?%]	Tsuchiya et al., 2008 case 4
	mar 1 = der(11)r(4; 11)(::11q11→11q12.1::4q12::)	
	mar 2 = der(7)(:p11.1:)	
	mar 3 = der(1)(:p12:)	
	mar 4 = der(X)(:p11.1→q11.1:)	
55	50,XY,+mar1,+mar2,+mar3,+mar4[100%] min(6)(:p11.1→q11.1:)	Fernández-Toral et al. (2010)
	min(8)(:p11.1→q11.1:)	
	min(11)(:p11.11→q11:)	
	min(12)(:p12.1→q10:)	
56	mos 50,XY,+mar1,+mar2,	Hochstenbach et al. (2013)
	+mar3,+mar4[5]/49,XY,+mar1,+mar2,	
	+mar3[99]/48,XY,+2mar[70]/47,XY,+1mar[22]/46,XY[3]	
	mar1 =	
	r(11)(::p11.12→q12.1::)	
	mar2 = r(12)(::p11.1→q11::)	
	mar3 = r(X)(::p11.1→q12::)	
	mar4 = ??	
	#11: arr[hg19] 11p12q12.1(50,713,402-56,738,678)x3	
	#12: arr[hg19] 12p11.1q11(34,436,391-34,589,410)x3	
	X: arr[hg19] Xq12(64,811,035- 64,818,437)x3	
57	mos 49-53,XY,+mar1-7[100%]	Ulmer et al. (1997)
	r(11) in ∼84%	
	?r(1) in ∼90%	
	?r(3) in ∼80% min(X) in ∼88%	
	min(20) in ∼74%	
	min(14) in ∼94%	
	min(21) in ∼83%	

**TABLE 2:** Overview of the eight cases from the literature with centromere 11-near imbalances similar to those induced by an sSMC(11)—for more details, see Supplementary Table S1; all molecular karyotyping data is given here in build GRCh37/hg19.

#	Karyotype	Ref
No sSMC(11) cases with centromere-near imbalances: no or minor clinical findings		
A	46,XX,der(11).ish dup(11)(p11.2q11.1)(RP11-397M16++,D11Z1++,RP11-77M17+)	Kieback et al. (2007)
B	46,XX,der(11)mat.ish dup(11)(p11.1q11)(D11Z1++)	Till et al. (1991)
**No sSMC(11) cases with centromere-near imbalances: with clinical findings**		
C	46,XX,dup(11)(p11.2p11.1)	Guichet (2005)
	LSI-FISH duplication size ∼6 Mb	
D	46,XY,dup(11)(p12)	Goossens et al. (1999)
E	46,XY,dup(11)(p12p11.2)mat arr[hg19] 11p12p11.2(40,231,033_50,762,504)x3	Chen et al. (2021)
F	46,XY,ins(11)(11; 11)(q14.5p14.1p11.2)	Strobel et al. (1980)
G	mos 46,XY,dup(11)(q11q13.3)[29]/46,XY[6]	Jehee et al. (2007)
	arr[hg18] 11q11q13.3(56,000,000_78,750,000)	
H	mos 46,XY,dup(11)(q12.1q13.3)[53%]/46,XY[47%]	Robberecht et al., 2012 case 3

### 2.2 Karyotype analyses and molecular cytogenetic studies

The 18 cases presented here were identified by standard banding cytogenetics (Hliscs et al., 1997) and the corresponding sSMCs were characterized as derived from chromosome 11 by centromere-specific multicolor-FISH (cenM-FISH) (Nietzel et al., 2001). These results were confirmed by a commercial centromere-11-specific probe: CEP 11 (D11Z1) (Vysis/Abbott, Düsseldorf, Germany) and the euchromatic content was further determined by a subcenM-FISH-probe set as previously reported, i.e., a partial chromosome paint for 11p and 11q, probes for 11p11.2 (RP11-397M16–hg19: 48,303,671-48,479,496) and 11q12 (RP11-77M17–hg19: 57,352,936-57,521,103) that were applied, together with D11Z1 (Starke et al., 2003). The identification of sSMCs by chromosomal microdissection and subsequent reverse hybridization was carried out as published by Weimer et al. (1999).

Array-comparative genomic hybridization (aCGH) was completed with microdissected material from the corresponding sSMC as previously described (Melo et al., 2011). In some cases evaluated in this manner, the results of sizing the sSMC were not as exact as when using genomic DNA; thus, the values were rounded to the second digit after the decimal point as Mb.

Molecular data from literature using other builds were translated into GRCh37/hg19 using the University of California Santa Cruz (UCSC) webpage using the function ‘View’ ‘In Other Genomes (Convert)’ and are included in Tables 3, 4. Data for Supplementary Table S4; Supplementary Figure S1 was acquired using the UCSC browser combined with OMIM or DECIPHER, Database of Genomic Variants, GnomAD, and localization of segmental duplications.

**TABLE 3:** Selected details on cases from Tables 1, 2 being suited to narrow down the triplo-insensitive regions of pericentric 11p and 11q. Data from cases providing the largest uncritical pericentric regions are highlighted in bold.

#	Region being trisomic (hg19)	Data
p-arm		
2	**48,303,671**-55,700,000	ish
A	**48,303,671**-55,700,000	ish
q-arm		
6	51,600,000-57,521,103	ish
7	51,600,000-59,319,390	aCGH and ish
1	51,095,992-**60,473,821**	aCGH

**TABLE 4:** Selected details on cases from Tables 1, 2 being suited to narrow down the borders between triplo-insensitive and sensitive regions of pericentric 11p and 11q. Data from cases providing the largest uncritical pericentric regions are highlighted in bold.

#	Region being trisomic (hg19)	Technique
p-arm		
10	**42,922,228**-50,768,675	aCGH
13	40,233,424-54,943,424	aCGH
E	40,231,033-50,762,504	aCGH
F	30,000,000-51,600,000	GTG
q-arm		
G	56,243,424-**79,072,352**	aCGH

## 3 Results

**FIGURE 1:**
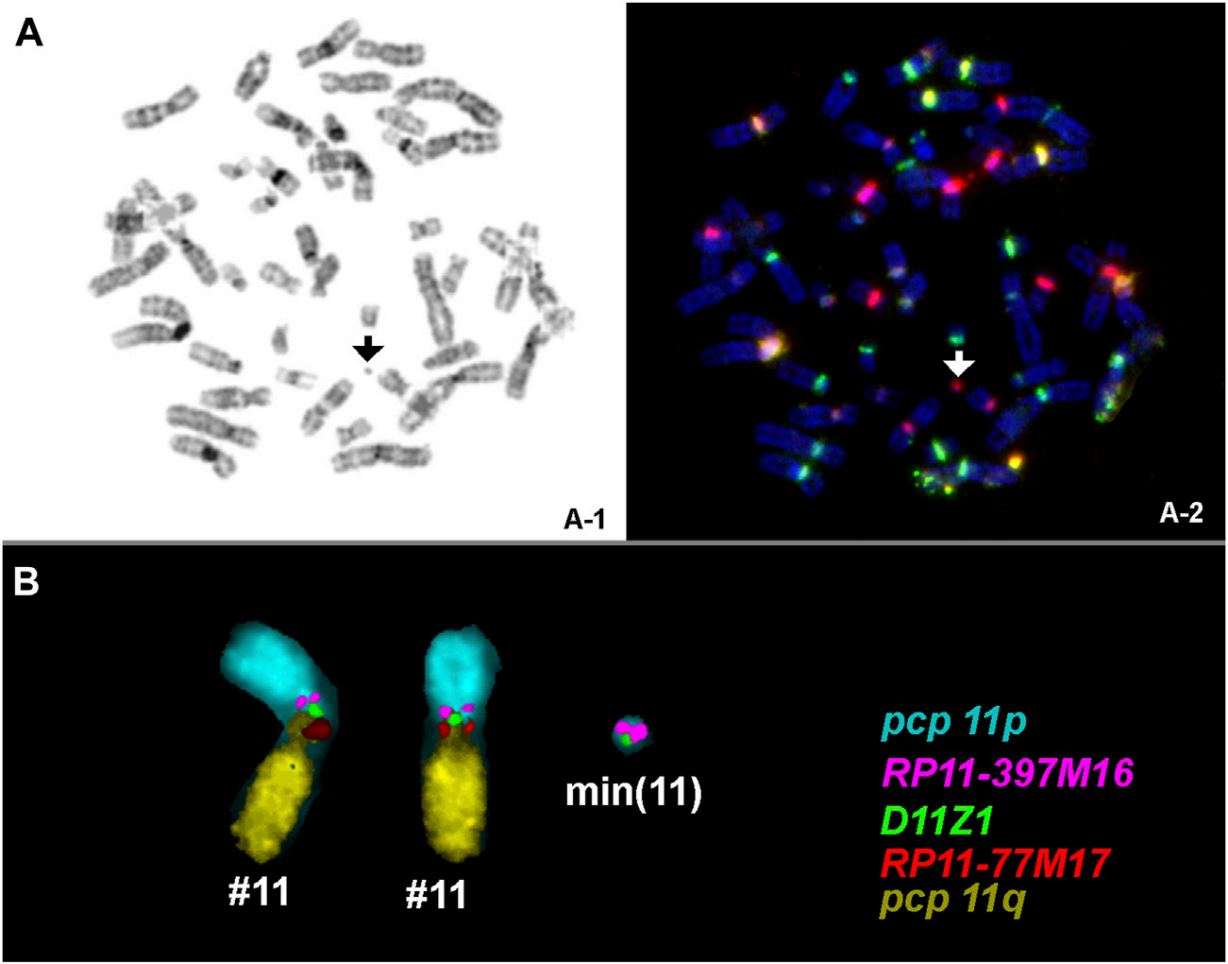
An sSMC was detected by GTG-banding **(A)** in all amnion cells studied; cenM-FISH identified chromosome-11-origin [arrowhead in **(B)**]; the presence of euchromatic content of chromosome 11p and the shape was determined by subcenM-FISH by applying the five probes as shown **(C)**. A min(11) (:p11.12→q11:) was characterized. Abbreviations: pcp, partial chromosome paint.

The 18 sSMC(11) cases presented herein were characterized for origin, shape, and content by cenM-FISH and subcenM-FISH; an example result is presented using case 2 (see Figure 1). The corresponding cases are in Table 1: cases 2-4, 6-7, 12, 15, 17, 24-26, 30, 33-36, 39, and 51. Together with the 39 cases from the literature, 57 sSMC(11) cases were available for analysis. In total, 12 cases were clinically normal (cases 1-9, 43-45) and 23 were clinically abnormal (cases 11-21, 46-57), and for the remaining 22, no clinical details were available and/or a clear clinical correlation to the presence of an sSMC was not possible. The male-to-female ratio for the 52/57 cases with known gender was 29:23.

In Table 1, all 57 cases are listed with genotype and size of the sSMC(11) and designated clinically normal (which may include infertility) or abnormal (for more details, see Supplementary Table S1).

**FIGURE 2:**
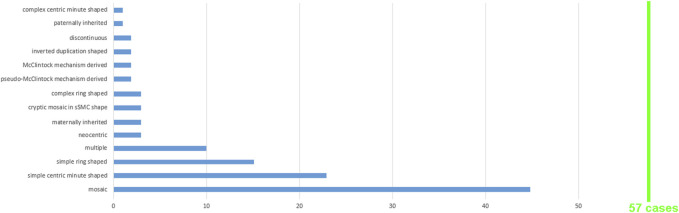
A total of 57 sSMC(11) cases were grouped according to the 14 features shown; for more details see Supplementary Table S3.

For 43 of the 57 cases, the shape was reported: 50% had min-, 47% had r-, and 3% had inv dup-shaped. The sSMC(11) group distribution is shown in Figure 2 with observation of all shape subtypes excluding UPD(11)-associated sSMCs; several sSMCs could be attributed to more than one group and can be observed two or more times in Supplementary Table S3, which serves as the basis for the data presented in Figure 2.

With the exception of case 41, all cases were constitutional sSMCs; case 41 was an acquired sSMC associated with atypical chronic myelogenous leukemia (aCML).

In Table 2, all eight cases from the literature with imbalances similar to those associated with sSMC(11) are listed with the genotype/size of the imbalance and clinical outcome (for more details, see Supplementary Table S2).

Only those cases from Tables 1, 2 with a good molecular(cyto)genetic characterization and/or a suitable clinical description were acceptable for inclusion in Tables 3–5. Similarly, cases with complex, discontinuous, and/or multiple sSMCs (11) could not be considered; sSMCs formed by (pseudo-)McClintock mechanism were also excluded.

Cases 1 to 9, A and B, demonstrated no relevant clinical signs associated with pericentric partial trisomy 11. Accordingly, Table 3 includes 5 cases (from 11 total clinically normal) that were suitable to determine the minimal pericentric triplo-insensitive region of chromosome 11; this region spans chromosome 11 positions 48,303,671 to 60,473,821 (hg19), at a minimum. This region comprises 305 genes, with only 11 OMIM morbid annotated genes, mostly activated by point mutations (Supplementary Table S4).

Cases 13-21 and C to H show relevant clinical signs most likely due to pericentric partial trisomy 11; Table 4 includes those five cases suitable to determine the region adjacent to the triplo-independent region (see Table 3). Thus, the triplo-sensitive region in 11p starts between 42,922,228-48,303,671, and the corresponding region in 11q starts between 60,473,821-79,072,352 Mb (hg19).

**FIGURE 3:**
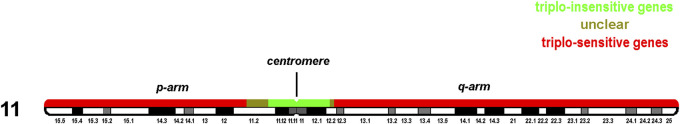
Regions containing dosage/duplication independent (green) and dependent genes (red); Olive stretches between those regions lack cases to narrow down the corresponding green and red regions.

To further refine the pericentric triplo-(in)sensitive regions of chromosome 11 ‘pathogenic’ and ‘likely pathogenic’ gains and duplication in the region were checked in OMIM for DECIPHER patients 411500 and 300792 (Supplementary Figure S1). Therefore, no pathogenic patient report is available for regions 45,048,321 to 61,479,322. In addition, population data for known benign copy number gains (Database of Genomic variants and gnomAD, records dgv1111n100 and nsv832175) also fit with data from Table 3 and DECIPHER data. Thus, the uncritical proximal triplo-insensitive region can be refined to 47,675,469 to 60,516,539 Mb, i.e., a region of 12.841,070 Mb (Supplementary Figure S1; Figure 3). However, the exact starting points of triplo-sensitive regions in 11p and 11q could only be narrowed to 45,048,321 to 47,675,469 Mb and 60,516,539 to 61,479,322, respectively; these are regions of 2.63 and 0.96 Mb.

Table 5 summarizes clinical signs and symptoms according to the nine cases (also based on data from Tables 3, 4) suitable for a preliminary genotype–phenotype correlation.

**TABLE 5:** Selected details on cases from Tables 1, 2 providing clinical data for a first genotype-phenotype correlation. Data from cases providing the largest uncritical pericentric regions are highlighted in bold.

Affected	Signs and symptoms	11p-cen-near (cases included from Tabs 1 and 2)	11p-cen-near (# of cases)	11q-cen-near (# of cases)	11q-cen-near (cases included from Tabs 1 and 2)
growth	growth retardation (prenatal and/or postnatal)	(case D)	1	1	(case 29)
head—eyes	blepharophimosis/ptosis		0	**1**	(case 15)
	strabism		0	**1**	(case 15)
head - face	cleft palate	(case F)	1	0	
	facial dysmorphism (no details given, or other than listed, i.e., unspecific ones)	(cases D, E, F)	3	4	(cases 15, G, H, 29)
heart	heart defect (not specified)		0	**2**	(cases G, 29)
mental	developmental delay	(cases 13, C, D, E, F)	5	4	(cases 15, G, H, 29)
	intellectual disability	(case D)	1	1	(case 15)
muscles	hypotonia	(case 13)	1	1	(case 29)
		5 cases		4 cases	

## 4 Discussion

This study adds 18 new sSMC(11) cases to the literature. Among the 57 cases evaluated herein, several types of sSMC shapes and subtypes are presented, as shown in Figure 2. Like non-acrocentric-derived sSMCs from other chromosomes, chromosome 11-derived sSMCs are predominantly r- and min-shaped, with very rare incidences of the inv dup-shaped (Liehr, 2012). The under- or over-representation of specific subgroups of sSMC(11) is likely attributable to the low case number availability. Accordingly, (i) no case with sSMC-associated UPD(11) is reported as of yet, (ii) 5/57 sSMC(11) cases (8.8%) were formed by (pseudo-)McClintock mechanism (Baldwin et al., 2008), which is > 8× more than observed among all sSMCs generally (Liehr, 2023), and (iii) 10/57 sSMC(11) (17.5%) were observed in multiple sSMC carriers in comparison to ∼1.2% observation among all sSMC(11)-carriers. The fact that ∼80% of sSMC(11) carriers show mosaic presentation aligns well with the observation that r- and min-shaped sSMCs are less mitotically stable (Hussein et al., 2014).

Clinical follow-up, particularly in prenatal cases is a problem (Detorri, 2011). As such, it is normal, that for 22/57 sSMC(11) cases (38.6%), no clinical data is available for analysis. Similarly, reliable data for parental origin was provided for only 4/55 cases, three of maternal origin and one of paternal origin; this aligns with the predominance of maternal sSMC inheritance (Dalprà et al., 2005). The sex of the sSMC(11) carriers was not reported in 5/57 cases (∼9%); a 1:1.26 male-to-female ratio does not seem remarkable for one or the other gender, as is established for all sSMCs (Liehr et al., 2011).

Unfortunately, limitations of sufficient clinical and molecular(cyto)genetic data negatively influenced the characterization and analysis of cases. For the 57 sSMC(11) cases summarized herein, these data were comprehensive for only six cases: 1, 2, 6, 7, 10, and 13 (see Tables 3, 4). Thus, to approach the goal of defining triplo-insensitive pericentric regions for chromosome 11 and provide the first preliminary genotype–phenotype correlation for 11p and 11q proximal trisomies, eight more cases from the literature were also included (Table 2), leading to four more cases (A, E, F, and G) in Tables 3, 4. These cases have similar centromere-near imbalances as induced by sSMC(11) from interstitial, intrachromosomal 11 duplications.

Euchromatic copy number gains, as induced by sSMC presence, are considered the main influencer of genotype–phenotype correlations (Liehr, 2012). However, there may be UPD of an sSMC sister-chromosome associated with disease causation even with a heterochromatic sSMC (Liehr, 2012). Finally, mosaicism has a major influence on genotype–phenotype correlation in these patients, which should be discussed, evaluated, and understood. For mosaicism, we must primarily consider that generally, only one to three tissues of the human body can be studied. In the prenatal evaluation, this is chorionic villus sampling, amniotic fluid, but may be fetal blood, too; postnatally, peripheral blood and buccal mucosa are easily accessible. The other ∼400 tissues of the human body remain a black box regarding the mosaic state of the sSMC. Studies have demonstrated that sSMCs seem to be present in all body tissues in different mosaic states (Fickelscher et al., 2007). Thus, it is not surprising that mosaicism levels generally have no major influence on the phenotype (Liehr, 2023). In cases where an sSMC is found in peripheral blood at 50% or more, if an sSMC is associated with a clinical impact, the patient will present with typical signs and symptoms. Considering this link, it seems justifiable to also include mosaic cases in a genotype–phenotype correlation study.

However, there are also rare exceptions, e.g., Pallister-Kilian syndrome patients, who are not found to have sSMC(12) in peripheral blood, unlike all other tissues (Liehr and Al-Rikabi, 2019). Furthermore, there can also be normal carriers of otherwise deleterious sSMCs, e.g., inv dup(9p) with the marker chromosome in nearly 100% of the peripheral blood cells (Liehr and Al-Rikabi, 2019).

For copy number variations (CNVs), it is well known that they may span up to several euchromatic, gene-containing megabases in size and can be placed into two groups based on current knowledge: 1) not associated with disease causation, and 2) associated with microdeletion/microduplication syndromes (Itsara et al., 2009). They are neither clearly distinguished by size nor by number of genes involved. The only difference established is that CNVs leading to the aforementioned syndromes contain at least one dosage-sensitive gene (Rice and McLysaght, 2017). The best example may be hereditary neuropathy with liability to pressure palsies (HNPP) and Charcot-Marie Tooth disease type 1A (CMT1A) associated with a 1.4 Mb microdeletion or microduplication in 17p11.2, respectively. The 1.4 Mb of DNA includes dozens of genes; however, only the *PMP22* gene is dosage-sensitive and results in the respective syndrome when present in one or three copies instead of two (van Paassen et al., 2014). Similarly, previous research provided evidence that pericentric CNVs/copy number gains induced by sSMC presence are comparable with either a harmless CNV or a microduplication syndrome (Qi et al., 2013; Liehr, 2023). Simply stated, the proximal centromere-near regions of all chromosomes seem to be no issue for human cells if they are present in more than two copies, as long as they only contain DNA/genes that are dosage insensitive. The main goal of a genotype–phenotype correlation of an sSMC must be to determine the regions, which are unproblematic if present in more than two copies. In the suggested region, 305 genes are included, which are obviously insensitive to copy numbers gains (Supplementary Table S4). Finally, the regions that contain the first centromere-near dosage-dependent or better triplo-sensitive gene(s) should be determined. The more informative sSMC cases that are available, the better such a link can be.

Even though only a few cases of chromosome 11 were available for such an undertaking, herein, a first refinement of clinical and molecular(cyto)genomic data was performed. As outlined in Tables 3, 4 (see also Supplementary Figure S1), the minimal pericentric region of chromosome 11 that does not contain dosage-sensitive, or more precisely, duplication-sensitive genes, is between positions 47.68 and 60.52 Mb (GRCh37/hg19). Furthermore, clinical symptoms are expected if an sSMC(11) contains greater than 2.63 Mb or 0.96 Mb of DNA for the short or long arm, respectively. Increasing the publication of more informative sSMC(11) cases will further delineate these regions.

Positing a preliminary first genotype–phenotype correlation of deleterious partial trisomy 11p compared to 11q, the data from only nine cases are presented in Table 5. As to be expected, clinically abnormal sSMC(11) cases demonstrate rather unspecific common symptoms like dysmorphism, developmental delay, intellectual disability, and hypotonia. Of interest, eye and heart problems were isolated to partial trisomy 11q cases only.

Overall, genotype–phenotype correlations for microduplication syndromes are hampered by clinical variance in the presentation of symptoms (Watson et al., 2014). This is also the case for sSMCs, which may also be more difficult to interpret due to mosaicism (Liehr and Al-Rikabi, 2019), UPD (Liehr et al., 2011), an insufficiently characterized sSMC (see Table 1), and/or the presence of a harmless sSMC with an uncharacterized variant in any human gene associated with disease causation (Nelle et al., 2010). Still, herein, the first steps needed to distinguish a harmful sSMC from a harmless sSMC(11) are presented.

## Data Availability

The datasets presented in this study can be found in online repositories. The names of the repository/repositories and accession number(s) can be found in the article/Supplementary Material.

## Supplementary material

Supplementary Figure S1 The pericentric region of chromosome 11 is shown in the UCSC genome browser. The orange rectangle indicates the triplo-insensitive region as determined in Table 3. The depicted Decipher track was filtered for “pathogenic” and “likely pathogenic” patients. The OMIM track only shows OMIM morbid annotated genes. The database of genomic variants and gnomAD were filtered for duplications/gains of >0.1 Mb in the population, which fits well with the concentration of segmental duplication (bottom track).
