# Supplementary figures and images for: Small supernumerary marker chromosomes derived from human chromosome 11
Source: Front Genet. 2023 Dec 15;14:1293652. doi: 10.3389/fgene.2023.1293652 (PMC10763568; doi:10.3389/fgene.2023.1293652)

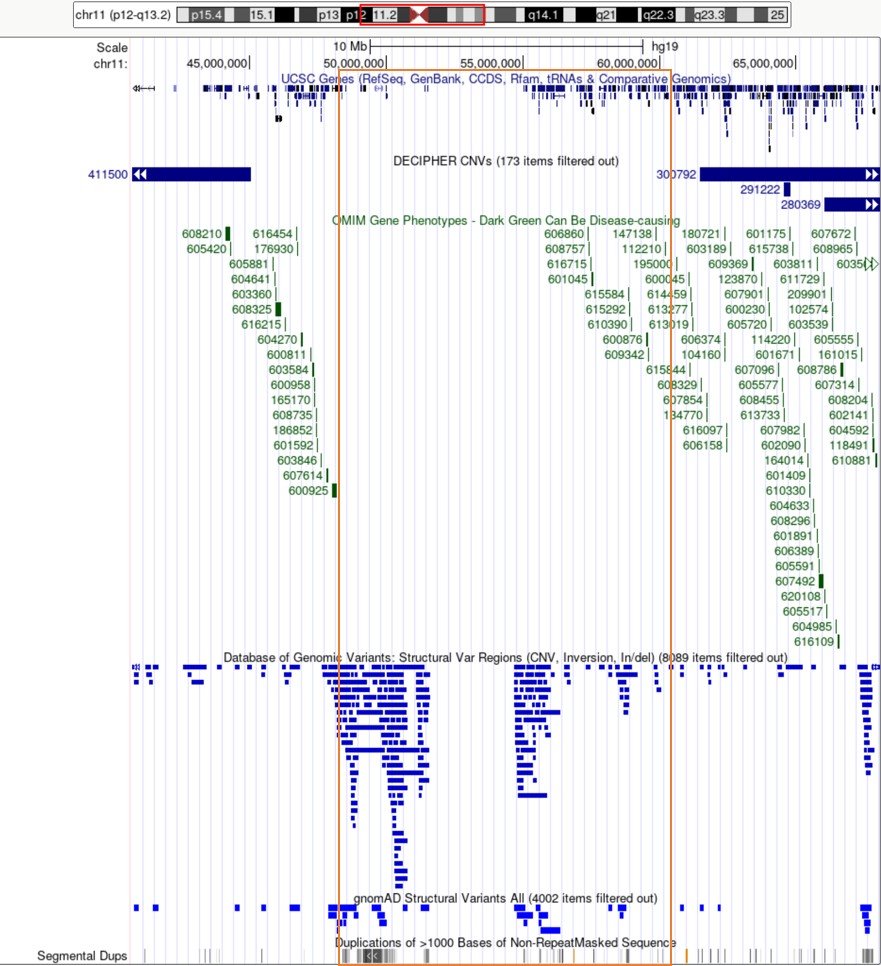

Supplement: Supplementary file 1 [file Image1.JPEG]
