# Supplementary material for: Small supernumerary marker chromosomes derived from human chromosome 11
Source: Front Genet. 2023 Dec 15;14:1293652. doi: 10.3389/fgene.2023.1293652 (PMC10763568; doi:10.3389/fgene.2023.1293652)
Supplement: Supplementary file 2 [file DataSheet1.docx]

Supplementary Material

# Supplementary Table 1

**Supplementary Table 1**

Cases with sSMC(11) reported in the literature and studied herein

Abbreviations (excluding ISCN nomenclature): aCGH = array- comparative genomic hybridization; Ad = adult; AF = amniotic fluid; AMA = advanced maternal age; ASD = artrial septum defect; BAC = bacterial artificial chromosome based; BM = bone marrow; cenM = centromere specific multicolor-FISH; cep = centromeric probe; CREST = acronym for calcinosis, Raynaud's phenomenon, esophageal dysfunction, sclerodactyly, and telangiectasia; DD = developmental delay; DYS = dysmporphisms; FISH = fluorescence in situ hybridization; IUGR = intrauterine growth retardation; LSI = locus-specific probe; m = months; Mb = megabasepair; MR = intellectual disability; n.a. = data not available; NGS = next generation sequencing, p-arm = short chromosome arm; PBL = peripheral blood lymphocytes; PN = prenatal; q-arm = long chromosome arm; SNP = single nucleotide polymorphism; sSMC = small supernumerary marker chromosome; subcenM = chromosome specific subcentromere specific multicolor-FISH probe set; TOP = termination of pregnancy; UPD = uniparental disomy; VSD = ventricle septum defect; w = weeks; wcp = whole chromosome paint; y = years; ? = postnatal – age of patient not known;

| **#** | **Case number acc. to**  **https://cs-tl.de/DB/CA/sSMC/0-Start.html** | **gender** | **age/ material studied** | **indication** | **karyotype** | **methods** | **Ref.** |
| --- | --- | --- | --- | --- | --- | --- | --- |
| **sSMC(11) cases: no clinical findings (excluding infertility)** | | | | | | | |
| 1 | 11-O-p11.2/1-1 | male | 40y / PBL | infertile | 47,XY,+r(11)(::p11.12→q12.2::).arr[hg18] 11q12.1-12.2(50,952,568_60,230,397)x3 | SNP-aCGH | Liehr, 2014 case 11-1 |
| 2 | 11-O-p11.2/2-1 | male | PN / AF | AMA, mother normal; sSMC in 10/23 cells | 47,XY,+mar mat.ish min(11)(:p11.12→q11:)(RP11-397M16+,D11Z1+,RP11-77M17-) | cenM, subcenM | this study |
| **#** | **Case number acc. to**  **https://cs-tl.de/DB/CA/sSMC/0-Start.html** | **gender** | **age/ material studied** | **indication** | **karyotype** | **methods** | **Ref.** |
| 3 | 11-O-p11.1/1-1 | male | PN / AF | AMA, child normal at 1 m | mos 47,XY,+mar[50%]/46,XY[50%].ish min(11)(:p11.1→q11:)(RP11-397M16-,D11Z1+,RP11-77M17-) | cenM, subcenM, UPD test (microsatellites) | this study |
| 4 | 11-O-p11.1/1-2 | male | PN / AF | AMA, child normal at 3 m | mos 47,XY,+mar[21]/46,XY[32].ish min(11)(:p11.1→q11:)(RP11-397M16-,D11Z1+,RP11-77M17-) | cenM, subcenM | this study |
| 5 | 11-O-p11.1/1-3 | female | PN / AF | AMA, child normal at 4 m | mos 47,XX,+mar[18]/46,XX[4].ish min(11)(:p11.1→q11:)(D11Z1+).arr[hg18] (X,1-22)x2 | spectral karyotyping; cep11-FISH, aCGH | Chen et al., 2017 |
| 6 | 11-O-p11.1/2-1 | female | 24y / PBL | Normal female, studied due to daughter with DD and sSMC(11) | 47,XX,+mar.ish  r(11)(::p11.1→q12.2::)[10]/r(11)(::p11.1→q12.2: :q12.2→p11.1::)[7]/min(:q12.2→p11.1::p11.1→q12.2:)[3] (RP11-397M16-,D11Z1+,RP11-77M17+) | cenM, subcenM, UPD test (microsatellites) | this study |
| 7 | 11-O-p11.1/3-1 | male | 39y / PBL | infertile | 47,XY,+mar.ish min(11)(:p11.1→q12.1:)(RP11-397M16-,D11Z1+,RP11-77M17+)  .arr[hg19] 11q12.1(55,896,790_59,319,390)x3 | cenM, subcenM, aCGH | this study |
| 8 | 11-CO-1 | female | 34y / PBL | normal;  father with 59y CREST syndrome | mos 47,XX,+mar pat[60%]/46,XX[40%].ish r(11)(D11Z1+) | cep11-FISH | Haaf et al. 1992 |
| 9 | 11-CO-2 | male | PN / AF | AMA, child normal at 3 m | mos 47,XY,+mar[32]/46,XY[12].ish r(11)(wcp11+) | wcp11-FISH | Kozlowski et al., 2006 case 26 |
| **#** | **Case number acc. to**  **https://cs-tl.de/DB/CA/sSMC/0-Start.html** | **gender** | **age/ material studied** | **indication** | **karyotype** | **methods** | **Ref.** |
| **sSMC(11) cases: with clinical findings** | | | | | | | |
| 10 | 11-W-p12/1-1 | n.a. | PN / AF | abnormal | mos 47,XN,+mar[74%]/46,XN[26%].arr[hg19] 11p12~11.2(42,922,228_50,768,675)x3 | aCGH | Joshi et al. 2019 case P10 |
| 11 | 11-W-p11.2/1-1 | female | ? / PBL | abnormal | mos 47,XX,+mar[53%]/46,XX[47%] aCGH data details not provided | aCGH | Neill et al. 2010 case 29361 |
| 12 | 11-W-p11.2/1-2 | male | PN / fibroblasts | missed abortion in 7+1 week of gestation | mos 47,XY,+mar[77%]/46,XY[23%].ish min or r(11)(:p11.2→q11.1:)(RP11-397M16+,D11Z1+,RP11-77M17-) | cenM, subcenM | this study |
| 13 | 11-W-p11.12/1-1 | male | 3y / PBL | at birth normal. postnatal psychomotor DD, muscular hypotonia | mos 47,XY,+mar[33%]/46,XY[67%].ish min(11)(:p12→q11:)(RP11-397M16+,D11Z1+,RP11-77M17-) .arr[hg18] 11p12(40,190,000_54,700,000)x3 | cenM; subcenM aCGH | Guilherme et al., 2012case Sm-5 |
| 14 | 11-W-p11.12/2-1 | n.a. | ? / PBL | hypotonia; gross motor delay; seizures; macrocephaly; intermittent exotropia | mos 47,XN,+mar[14%]/46,XN[86%]  most likely a r(11)(::p11.12→q12.1::); no clear data for aCGH - only given: size on sSMC p-arm 0.2 MB and q-arm 2.3 MB | kind of subcenM-FISH; aCGH | Baldwin et al., 2008 case 13 |
| **#** | **Case number acc. to**  **https://cs-tl.de/DB/CA/sSMC/0-Start.html** | **gender** | **age/ material studied** | **indication** | **karyotype** | **methods** | **Ref.** |
| 15 | 11-W-p11.12/3-1 | female | 13y | at 13 years: facial DYS, strabismus, ptosis, discrete MR and DD. However, mother with 36 years: no apparent MR; congenital cardiopathy (corrected with surgery) | mos 47,XX,+mar mat[70%]/46,XX[30%].ish r(11)(::p11.12→q13.1::)[6]/ r(11;11)(::p11.12→q13.1: :p11.12→q13.1::)[3]/ min(11)(:p11.12→q13.1:)[4]  (RP11-397M16+,D11Z1+,RP11-77M17+)  .arr[hg18] 11p11.12q13.1(50,470,000_65,020,000)x3 | cenM, subcenM, aCGH, UPD-test (microsatellites) | this study |
| 16 | 11-W-p11.12/3-2 | male | ? / PBL | twin pregnancy - other twin normal; at birth all values at 3^rd^ centile; at 15 months normal growth parameters; but psychomotor retardation and DYS | mos 47,XY,+mar[86]/46,XY[14].ish r(11)(::p11.12→q13.1::)(RP11-318O24+,RP11-100E23+,CTD-3202L3+,RP11-720L5+)  no clear data for aCGH - only given: size on sSMC p-arm ~1.5 Mb and q-arm 10.04 Mb | kind of subcenM-FISH; aCGH | Castronovo et al., 2013 case 5 |
| 17 | 11-W-p11.12/4-1 | male | PN / AF | AMA; child spontaneously aborted 22 week of gestation | 47,XY,+mar[100%].ish min(11)(:p11.2→q11::q11→p11.2:)(RP11-397M16++,D11Z1+,RP11-77M17-) | cenM, subcenM, UPD-test (microsatellites) | this study |
| **#** | **Case number acc. to**  **https://cs-tl.de/DB/CA/sSMC/0-Start.html** | **gender** | **age/ material studied** | **indication** | **karyotype** | **methods** | **Ref.** |
| 18 | 11-CW-1 | female | 4y / PBL | micrognathia; DYS, DD. At 4.8y growth parameters at 3^rd^ centile; DYS | mos 47,XX,+mar[?]/46,XX[?].ish min(11)(D11Z1+) | cep11-FISH | Rauch et al., 1992 case 7 |
| 19 | 11-CW-2 | male | PN, 4m / AF, PBL | at birth: cleft soft palate, VSD, wet lung; hypertonia and short statures at age of 7m; minor DYS; DD and moderately retarded at 4y; | AF: mos 47,XY,+mar[52%]/46,XY[48%] PBL at birth: 46,XY [200] PBL at 4m: mos 47,XY,+mar[36%]/46,XY[64%]  .ish r(11)(D11Z1+)  no data for aCGH provided | all ceps in FISH, aCGH | Daniel and Malafiej 2003 case 7 |
| 20 | 11-CW-3 | male | ? / PBL | mild psychomotor delay | mos 48,XY,+mar1,+mar2[?]/47,XY,+mar1[?]/47,XY,+mar2[?]/46,XY[?]  .ish mar(11)(D11Z1+) | different centromere probes in FISH | Sanz et al., 2005 case 4 |
| 21 | 11-CW-4 | female | 4y / PBL | intellectually disability, mild DYS, macrocephaly | mos 47,XY,+mar[70%]/46,XY[30%]  no clear data for aCGH - only given: size 5.9 Mb | aCGH | Silveira-Santos et al., 2012 |
| **sSMC(11) cases without clinical details / clear clinical correlation** | | | | | | | |
| 22 | 11-U-3 | male | PN /AF | AMA, TOP | mos 47,XY,+mar[31]/46,XY[5].ish r(11)(::p11→q12::)(D11Z1+) | cep11-FISH | Leung et al., 2004 case 4 |
| 23 | 11-U-5 | n.a. | n.a. | n.a. | 47,XN+mar[?%].ish mar(11)(D11Z1+) | cep11-FISH | Sanz et al., 2005 1 case |
| **#** | **Case number acc. to**  **https://cs-tl.de/DB/CA/sSMC/0-Start.html** | **gender** | **age/ material studied** | **indication** | **karyotype** | **methods** | **Ref.** |
| 24 | 11-U-7 | n.a. | PN / AF | AMA, n.a. | mos 47,XN+mar[50%]/46,XN[50%].ish r(11)(::p11→q12::)[3]/ r(11;11)(::p11→q12::p11→q12::)[2](RP11-397M16-,D11Z1+,RP11-77M17+) | cenM, subcenM | this study |
| 25 | 11-U-8 | n.a. | PN / AF | AMA, n.a. | mos 47,XN,+mar(11)[70%]/46,XN[30%].ish min(11)(:p11.1→q11:)(RP11-397M16-,D11Z1+,RP11-77M17-) | cenM, subcenM | this study |
| 26 | 11-U-10 | female | 6y / PBL | DD | 47,XX,+mar[100%].ish min(11)(:p11.1→q11:)(RP11-397M16-,D11Z1+,RP11-77M17-) | cenM, subcenM | this study |
| 27 | 11-U-11 | female | PN / AF | AMA, n.a. | mos 47,XX,+mar[21]/46,XX[30].arr[hg18] 11p12(43,085,000_51,400,000)x3 | aCGH | data provided by Dr. Joleen Viront, Akron, OH, USA |
| 28 | 11-U-14 | male | PN / AF | IUGR; spontaneous abortion in week 20 | mos 47,XY,+mar(11)[10]/46,XY[5] | FISH – no details | Thangavelu et al., 2011 |
| 29 | 11-U-15 | female | PN / AF | AMA, birth weight & growth at <3^rd^ centile. VSD; later good gain of weight & DD with muscu-lar hypotonia; large occiput, minor DYS | mos 47,XX,+mar[60-90%]/46,XX[10-40%].ish  min(11)(:p11.21→q13.1:)(RP11-397M16+,D11Z1+,RP11-77M17+) .arr[hg19] 11p11.21q13.1(49,850,000_64,600,000)x3 | cenM, subcenM, aCGH | Hamid et al. 2012 case 1 |
| **#** | **Case number acc. to**  **https://cs-tl.de/DB/CA/sSMC/0-Start.html** | **gender** | **age/ material studied** | **indication** | **karyotype** | **methods** | **Ref.** |
| 30 | 11-U-16 | male | PN / AF | AMA, n.a. | mos 47,XY,+mar[15]/46,XY[17].ish min(11)(:p11.?1→q1?1:)(RP11-397M16-,D11Z1+,RP11-77M17-) | cenM, subcenM | this study |
| 31 | 11-U-17 | female | PN / AF | AMA, TOP | mos 47,XX,+mar[94]/46,XX[53].arr[hg18] 11q12.1q12.3(55,509,438_62,106,928)x3 | aCGH | Marle et al., 2014 case 13 |
| 32 | 11-U-18 | female | PN / AF | AMA, n.a. | mos 47,XX,+mar[33]/46,XX[25].arr[hg18] 11p13q12.1(34,890,001_56,410,001)x3 | aCGH | Malvestiti et al., 2014 case AF-11 |
| 33 | 11-U-19 | female | PN / AF | AMA, enhanced nuchal translucency, n.a. | mos 47,XX,+mar[71]/46,XX[22].ish min(11)(:p11.1→q12.1:)[3]/ r(11)(::p11.2→q12.1::)[2]/r(11;11)(::p11.2→q12.1::p11.2→q12.1::)[3](RP11-397M16+,D11Z1+,RP11-77M17+) | cenM, subcenM | this study |
| 34 | 11-U-20 | male | PN / AF | AMA, n.a. | mos 47,XY,+mar[2]/46,XY[48].ish min(11)(:p11.11→q11:)(RP11-397M16-,D11Z1+,RP11-77M17-) | cenM, subcenM | this study |
| 35 | 11-U-21 | male | PN / AF | TOP | mos 47,XY,+mar[20%]/46,XY[80%].arr[hg19] 11p14.q12.1(30,800,000_56,650,000)x3 | cep11-FISH, aCGH | this study |
| 36 | 11-U-22 | female | PN / AF | AMA, n.a. | mos 47,XX,+mar[38]/46,XX[12].ish min(11)(:p11.11→q11:)(RP11-397M16-,D11Z1+,RP11-77M17-) | cenM, subcenM | this study |
| 37 | 11-U-23 | female | PN / AF | TOP | mos 47,XX,+mar[73]/46,XX[12].arr[hg19] 11p12.1q13.2(55,084,040_66,490,712)x3 | aCGH | Huang et al., 2019 case 16/16 |
| **#** | **Case number acc. to**  **https://cs-tl.de/DB/CA/sSMC/0-Start.html** | **gender** | **age/ material studied** | **indication** | **karyotype** | **methods** | **Ref.** |
| 38 | 11-U-24 |  |  |  | mos 47,XY,+min[15%]/46,XY[85%]  arr[hg19] 11p14q12.1(30,796,545_56,649,983)x3 | aCGH | Kontodiou et al., 2018 |
| **Complex sSMC(11) cases without clinical details / clear clinical correlation** | | | | | | | |
| 39 | 11-Uc-1 | female | PN / AF | AMA, no US-abnormalities, pregnancy continued - patient lost during follow-up | mos 47,XX,+mar dn[13]/46,XX[10].rev ish r(11)t(11;20)(::11p11.1→11q12.1::20q13.1?2→q13.32::) | Microdissection and reverse FISH, subcenM, UPD-test (microsatellites) | this study |
| 40 | 11-Uc-2 | male | ? / PBL | MR and DD, DYS; paternal balanced t(11;13)(q25;q14) | 47,XY,t(11;13)(q25;q14),+der(11)t(11;13)(q25;q14) | only banding cytogenetics | Metay et al., 2011 |
| **Discontinuous sSMC(11) cases without clinical details / clear clinical correlation** | | | | | | | |
| 41 | 11-Ud-1 | male | 47y / BM | atypical chronic myelogenous leukemia (CML); sSMC only in bone marrow, not in peripheral blood | mos 47,XY,+mar[18]/46,XY[2] r(11)(::p11.2→q13.1: :q14::).rev ish r(11)(::p11.2→q12.3::q14::)  .arr(hg18) 11p11.2q12.3(42.070,000_60,600,000)x3 | microdissection and reverse FISH, subcenM, aCGH | Starke et al., 2001 |
| **#** | **Case number acc. to**  **https://cs-tl.de/DB/CA/sSMC/0-Start.html** | **gender** | **age/ material studied** | **indication** | **karyotype** | **methods** | **Ref.** |
| 42 | 11-Ud-2 | male | PN / AF, CVS | Ultrasound: very mild decrease in growth parameters, TOP | AF: mos 47,XY,+mar[13]/46,XY[1] CVS: mos 47,XY,+mar[31]/46,XY[3]  seq[GRCh37] r(11)(::p11.2→q12.1::q12.1→q12.1::p15.5→p15.5::p15.4→p15.4::p11.2→p11.2::q12.1→q12.1::) chr11:g[47963807_cen_57123447inv::34232223_34232229::34232469_34232519::CACAGCTATGAGA::57123447_chr11:57150478: :TTTCCATTCCA::chr11:1791532_chr11:1831828::chr11:3681909_chr11:3826675::AGAGATGGAGCAAGCAATAGCAACTGCATA: :chr11:45940475_45998725::CACTGTAAATTGGG::47277430_47429775inv::chr11:57151476_57152981inv] chr11:g[57452438_57453327::57150508_57151481inv::57451445_57452437::57276408_57278946::18428101_18558839::57278947_57297284inv] | NGS | Kurtas et al., 2019 case sSMC11 |
| **sSMC(11) formed by McClintock mechansism** | | | | | | | |
| 43 | McCl-11-N-p11.2/1-1 | female | 36y / PBL | Normal herself, but abnormal fetus with del(11) only | mos 47,XX,del(11)(p14.3p11.2),+r(11)(::p14.3→  neo→ 11.12::)[16]/46,XX,del(11)(p.14.3p11.2)[4] | SNP-aCGH without details provided | Wang et al., 2023 |
| 44 | McCl-11-N-p14.3/1-1 | female | Ad / PBL | Normal herself, but abnormal child with del(11) only | 47,XX,del(11)(p11.12p11.2),+r(11)(::p11.12→p11.2::)[100%] | only banding cytogenetics | Chuang et al., 2005 |
| **sSMC(11) formed by pseudo-McClintock mechansism (neocentric)** | | | | | | | |
| 45 | PsMcCl-11-N-q22/1-1 | male | 1w / PBL | DD, DYS | 47,XY,del(11)(q22),+inv dup(11)(qter→q22::q22→qter**)**[100%] | cep probes, telomeric, wcps, LSI | Amor and Choo, 2002 |
| **#** | **Case number acc. to**  **https://cs-tl.de/DB/CA/sSMC/0-Start.html** | **gender** | **age/ material studied** | **indication** | **karyotype** | **methods** | **Ref.** |
| **sSMC(11) in multiple sSMC carriers** | | | | | | | |
| 46 | mult 2-10 | female | 5y / PBL | Hypotonic at birth; later DYS, ASD, hypoplasia of the left kidney. Neurologically, nystagmus in combination with divergent strabismus DD | 48,XX,+mar1,+mar2[16%]/ 47,XX,+mar1[26%]/ 47,XX,+mar2[22%%] 46,XX[36%]  mar1 = ish min(6)(:p11.2→q12:)(wcp6+,D6Z1+);  mar2 = min(11)(:p11.11→q11:)(D11Z1+,wcp11-) | spectral karyotyping; LSI-probes | Haddad et al., 1998 |
| 47 | mult 2-16 | male | ? / PBL | mild psychomotor delay | 48,XY,+mar1,+mar2[?]/ 47,XY,+mar1[?]/ 47,XY,+mar2[?]/ 46,XY[?]  mar 1: ish mar(11)(D11Z1+), mar2: ? | cep-FISH | Plattner et al., 1993 |
| 48 | mult 2-27 | n.a. | ? / | multiple congenital abnormalities | 48,+mar1,+mar2[36%]/ 47,+mar1[36%]/ 47,+mar2[28%]  mar1: arr[hg18] min(11)(:p10→q12.1:)(RP11-736I10+) mar2: arr[hg18] min(17)(:p11.2→q10:)(RP11-64J19+) | BAC-aCGH  distal clone given | Ballif et al., 2007 case 6 |
| 49 | mult 2-40 | male | PN / CH | AMA, normal sonography; previous pregnancy with trisomy 21 | 47,XX,+mar1[60%]/46,XX[40%] second sSMC not detected in cytogenetics but in FISH  final karyotype: 48,XY,+r(4)(::p14→q12::), +min(11)(:p11.11→q11:)[5]/48,XY,+r(4;4)(::p14→q12::p14→q12::),+min(11)(:p11.11→q11:)[5]/47,XY,+min(11)(:p11.11→q11:)[10] | cenM, subcenM | this study |
| **#** | **Case number acc. to**  **https://cs-tl.de/DB/CA/sSMC/0-Start.html** | **gender** | **age/ material studied** | **indication** | **karyotype** | **methods** | **Ref.** |
| 50 | mult 3-5 | male | 4m / PBL | Pierre-Robin-sequence, VSD, patent foramen ovale, cryptochism, flaccid joints, gothic palate, umbilical hernia, at birth urinary tract infection | 49,XY,+3mar[13]/48,XY,+2mar[22]/47,XY,+mar[23]/46,XY[2]  r(4)(::p12→q12::)[?] min(8)(:p11.21→q11.21::)[6] min(8)(:p21.1→p12: :p11.21→q11.21:)[14] 40.08-53.56 MB (hg19) r(11)(::p11.12→q11.1::)[?] acc. to NGS sSMC(8) is a mar(8)(:p11.21→q11.23: :q12.1q12::q12q12:) spanning chr8:g[:53561524::GCCCTAAGGAATCTCC: :60002688_60002774::TGG: :55759348_55759565: :TGATGTGTCACCTTGCTTTTAGATCTGAAGGTGA: :40082798:] | NGS | Kurtas et al., 2019 case sSMC8a |
| 51 | mult 4-12 | male | NB / PBL | IUGR; oligohydramnion; birth weight at 23^rd^ centile, lenght at 52^nd^ , OFC at 36^th^ ; at 3m: hypersomnia. pneumonia, hypotonia, dysmorphic face and others | 50,XY,+mar1, +mar2, +mar3, +mar4[16]/49,XY,+mar1, +mar2, +mar3[28]/48,XY,+mar1,+mar2[48]/47,XY,+mar1[8]  mar 1 = min(19) mar 2 = min(11) mar 3 = mar1 mar 4 = der(11;19) #11: arr[hg18] 11p11.12q12.1(48845776-58751035)x3 #19: arr[hg18] 19p12q12(23364013-34857827  possibly r(11;19): arr[hg18] 11q11q12.1(55,140,785-58,610,968)x3,19p12q12(23,967,466-24,006,230; 34,272,160-34,429,875)x3 | cep-FISH  aCGH | Fei et al., 2011 1 case |
| **#** | **Case number acc. to**  **https://cs-tl.de/DB/CA/sSMC/0-Start.html** | **gender** | **age/ material studied** | **indication** | **karyotype** | **methods** | **Ref.** |
| 52 | mult 4-4 | female | PN / AF | prenatal: bilateral cleft lip anomaly, ventriculomegaly, and possible agenesis of the corpus callosum. At birth, bilateral cleft lip anomaly, DYS. brain abnormalities  patent ductus arteriosus | 51,XX,+5mar[?%]/50,XX,+4mar[majority]/49,XX,+3mar[?%]/48,XX,+2mar[?%]  mar 1 = der(11)r(4;11)(::11q11→11q12.1::4q12::) mar 2 = der(7)(:p11.1:) mar 3 = der(1)(:p12:) mar 4 = der(X)(:p11.1→q11.1:) | spectral karyotyping  aCGH | Tsuchiya et al., 2008 case 4 |
| 53 | mult 4-5 | male | 30y / PBL | macrosomy & bilateral cryptorchidism. weight & length were clearly delayed (9m). DD, bilateral convergent strabismus. Learning diff-iculties, normal social behavior. Multiple hyperpigmented nevi all over his body | 50,XY,+mar1,+mar2,+mar3,+mar4[100%]  min(6)(:p11.1→q11.1:) min(8)(:p11.1→q11.1:) min(11)(:p11.11→q11:) min(12)(:p12.1→q10:) | cenM, subcenM | Fernández-Toral et al., 2010 |
| **#** | **Case number acc. to**  **https://cs-tl.de/DB/CA/sSMC/0-Start.html** | **gender** | **age/ material studied** | **indication** | **karyotype** | **methods** | **Ref.** |
| 54 | mult 4-9 | male | 24y / PBL | mild intellectual delay; obesity from 7y of age on; ASD, dysplastic aortic and pulmonary valves, club foot left, bilateral inguinal hernias, unilat. cryptochidism, assymm. growth of lower legs | 50,XY,+mar1,+mar2, +mar3,+mar4[5]/ 49,XY,+mar1,+mar2, +mar3[99]/ 48,XY,+2mar[70]/ 47,XY,+1mar[22]/ 46,XY[3]  mar1 = r(11)(::p11.12→q12.1::) mar2 = r(12)(::p11.1→q11::) mar3 = r(X)(::p11.1→q12::) mar4 = ?? #11: arr[hg19] 11p12q12.1(50,713,402-56,738,678)x3 #12: arr[hg19] 12p11.1q11(34,436,391-34,589,410)x3 X: arr[hg19] Xq12(64,811,035- 64,818,437)x3 | Ceps, aCGH, UPD test (microsatellites) | Hochstenbach et al., 2013 |
| 55 | mult 7-1 | male | PN / AF | suggested bladder outlet obstruction in ultrasound; child born in 36. week with 3190g, 52cm, OFC 35.3cm; Apgar 9/10/10; mild macrocephaly, unilateral hydro-nephrosis, at 4m (psycho)motor development normal | 49-53,XY,+mar1-7[100%]  r(11) in ~84% ?r(1) in ~90% ?r(3) in ~80% min(X) in ~88% min(20) in ~74% min(14) in ~94% min(21) in ~83% | microdissection and reverse FISH  all ceps  wcps | Ulmer et al., 1997 |

# Supplementary Table 2

Cases with similar imbalances to sSMC(11) reported in the literature. – for abbreviations see Supplementary Table 1.

| **#** | **Case number from Ref** | **gender** | **age/ material studied** | **indication** | **karyotype** | **methods** | **Ref.** |
| --- | --- | --- | --- | --- | --- | --- | --- |
| **No sSMC(11) cases with centromere-near imbalances: no or minor clinical findings** | | | | | | | |
| A | 11-O-IMB-p11.2/1-1 | female | PN / AF | ?AMA, normal at 2 y | 46,XX,der(11).ish dup(11)(p11.2q11.1)(RP11-397M16++,D11Z1++,RP11-77M17+) | subcenM | Kieback et al., 2007 |
| B | 11-O-IMBp11.11/1-1 | female | 39y / PBL | AMA, mother and child normal | 46,XX,der(11)mat.ish dup(11)(p11.1q11)(D11Z1++) | cep11-FISH | Till et al., 1991 |
| **No sSMC(11) cases with centromere-near imbalances: with clinical findings** | | | | | | | |
| C | 11-O-IMB-p11.2/2-1 | female | 15y / PBL | normal apart from isolated learning disability, particularly dyscalculia | 46,XX,dup(11)(p11.2p11.1) LSI-FISH duplication size ~6Mb | wcp 11; LSI probes (not specified) | Guichet 2005 |
| D | 11-W-IMB-p12/1-1 | male | 53y / PBL | mild to moderate MR, short stature, discrete DYS | 46,XY,dup(11)(p12) | only banding cytogenetics | Goossens et al., 1999 |
| E | 11-W-IMB-p12/2-1 | male | NB / PBL | DYS, DD, mother considered as normal | 46,XY,dup(11)(p12p11.2)mat arr[hg19] 11p12p11.2(40,231,033_50,762,504)x3 | aCGH | Chen et al., 2021 |
| F | 11-W-IMB-p11.2/1-1 | male | 5m / PBL | macular dysfunction, cleft lip and palate, and DD | 46,XY,ins(11)(11;11)(q14.5p14.1p11.2) | only banding cytogenetics | Strobel et al., 1980 |
| **#** | **Case number from Ref** | **gender** | **age/ material studied** | **indication** | **karyotype** | **methods** | **Ref.** |
| G | 11-W-IMB-q11/1-1 | male | 5m / PBL | multiple craniosynostoses, congenital heart defect and DD | mos 46,XY,dup(11)(q11q13.3)[29]/46,XY[6]  aCGH [hg18]: 56,000,000-78,750,000 | BAC-aCGH; different LSI probes in FISH | Jehee et al., 2007 |
| H | 11-W-IMB-q11/2-1 | male | ? / PBL | DYS | mos 46,XY,dup(11)(q12.1q13.3)[53%]/46,XY[47%] | only banding cytogenetics | Robberecht et al., 2012 case 3 |

# Supplementary Table 3

sSMC(11) cases from Supplementary Table 1 fall into 14 sSMC groups as shown here

| **sSMC group** | **Case numbers from Suppl. Table 1** |
| --- | --- |
| simple centric minute shaped | 2, 3, 4, 5, 7, 13, 15, 18, 25, 26, 29, 30, 33, 34, 36, 38, 41, 42, 46, 48, 49, 53 |
| simple ring shaped | 1, 6, 8, 9, 14, 16, 19, 22, 24, 43, 44, 50, 51, 54, 55 |
| complex centric minute shaped | 40 |
| complex ring shaped | 39, 51, 52 |
| inverted duplication shaped | 45 |
| multiple | 46, 47, 48, 49, 50, 51, 52, 53, 54, 55 |
| McClintock mechanism derived | 43, 44 |
| pseudo-McClintock mechanism derived | 45 |
| neocentric | 43, 44, 45 |
| mosaic | 3, 4, 5, 8, 9, 10, 11, 12, 13, 14, 15, 16, 18, 19, 20, 21, 22, 24, 25, 27, 28, 29, 30, 31, 32, 33, 34, 35, 36, 37, 38, 39, 41, 42, 43, 45, 46, 47, 48, 49, 50, 51, 52, 54, 55 |
| cryptic mosaic in sSMC shape | 6, 15, 33 |
| discontinuous | 41, 42 |
| maternally inherited | 2, 15 |
| paternally inherited | 8 |
| no corresponding information could be included for | 17, 23 |

# Supplementary Table 4

Of the 305 genes in the pericentric dosage insensitive region of chromosome 11 (chr11:48,303,671-60,473,821 Mb (hg19)), only 11 are OMIM morbid annotated genes, which are listed here.

| Gene | Trait | OMIM Morbid | Phenotype |
| --- | --- | --- | --- |
| *CBLIF*  cobalamin binding intrinsic factor  *609342 | autosomal recessive | Intrinsic factor deficiency  #26100 | vitamin B12 deficiency, sensory impairment, paresthesias, peripheral neuropathy, megaloblastic anemia |
| *CLP1*  cleavage factor polyribonucleotide kinase subunit 1  *608757 | autosomal recessive | Pontocerebellar hypoplasia type 10 (PCH10)  #615803 | neurodevelopmental and neurodegenerative disorder, severely delayed psychomotor development, progressive microcephaly, spasticity, seizures, brain atrophy, delayed myelination, some patients have dysmorphic features and an axonal sensorimotor neuropathy |
| *CTNND1*  catenin delta 1  *601045 | autosomal  dominant | Blepharocheilodontic syndrome-2 (BCDS2)  #617681 | eyelid anomalies, facial dysmorphism with hypertelorism, flat face, high forehead, conical teeth and tooth agenesis, cleft lip and palate, hair anomalies, hypothyroidism |
| *FAM111A*  FAM111 trypsin like peptidase A  *615292 | autosomal  dominant | Gracile bone dysplasia (GCLEB)  #602361 | perinatally lethal, gracile bones with thin diaphyses, premature closure of basal cranial sutures, microphthalmia |
|  |  | Kenny-Caffey syndrome (KCS2)  #127000 | severe proportionate short stature, cortical thickening, medullary stenosis of the tubular bones, delayed closure of the anterior fontanel, eye abnormalities, transient hypocalcemia, normal intelligence |
| *FAM111B*  FAM111 trypsin like peptidase B  *615584 | autosomal  dominant | Poikiloderma with tendon contractures, myopathy, and pulmonary fibrosis (POIKTMP)  #615704 | mottled pigmentation, telangiectasia, epidermal atrophy, can be accompanied by tendon contractures, myopathy, and progressive pulmonary fibrosis resulting in progressive dyspnea, heat intolerance, reduced sweating, thin hair |
| Gene | Trait | OMIM Morbid | Phenotype |
| *MPEG1*  macrophage expressed 1  *610390 | autosomal  dominant | Immunodeficiency-77 (IMD77)  #619223 | recurrent and persistent polymicrobial infections with multiple unusual organisms, skin and pulmonary infections are the most common |
| *MS4A1*  membrane spanning 4-domains A1  *112210 | autosomal recessive | Common variable immunodeficiency (CVID)  #613495 | recurrent respiratory infections, reduced numbers of memory B cells, defective antibody production, hypogammaglobulinemia |
| *MS4A2*  membrane spanning 4-domains A2  *147138 | autosomal  dominant | Susceptibility to atopy  #147050 | atopic hypersensitivity, asthma, eczema |
| *SERPING1*  serpin family G member 1  *606860 | autosomal recessive/ autosomal  dominant | Angioedema-1 and -2 (HAE1 and HAE2)  #106100 | clinically indistinguishable disorders, both episodic local subcutaneous/submucosal edema of the upper respiratory and gastrointestinal tracts, HAE1 (85% of patients, serum level of C1NH < 35%), HAE2 (normal C1NH levels of nonfunctional protein) |
|  | autosomal  dominant | Partial deficiency of complement component 4  #120790 | systemic lupus erythematosus |
| *STX3*  syntaxin 3  *600876 | autosomal recessive | Diarrhea 12, with microvillus atrophy (DIAR12)  #619445 | congenital enteropathy, severe dehydration, metabolic acidosis, dependent on total parenteral nutrition, loss of brush-border microvilli, microvillus inclusions, accumulation of subapical vesicles in villus enterocytes |
|  |  | Retinal dystrophy and microvillus inclusion disease (RDMVID)  #619446 | early-onset severe retinal dystrophy, intractable congenital diarrhea requiring total parenteral nutrition, loss of microvilli, microvillus inclusions, and accumulation of subapical vesicles in epithelial cells |
| *TMX2* thioredoxin related transmembrane protein 2 *616715 | autosomal recessive | Neurodevelopm disorder ,microcephaly, cortical mal- formations, + spasticity (NEDMCMS) #618730 | severe to profound global developmental delay, early-onset seizures, microcephaly, polymicrogyria, cerebral atrophy, unable to walk or speak, profoundly impaired intellectual development, axial hypotonia, peripheral spasticity |
